# Neuroprotective Function of 14-3-3 Proteins in Neurodegeneration

**DOI:** 10.1155/2013/564534

**Published:** 2013-12-02

**Authors:** Tadayuki Shimada, Alyson E. Fournier, Kanato Yamagata

**Affiliations:** ^1^Neural Plasticity Project, Tokyo Metropolitan Institute of Medical Science, 2-1-6 Kamikitazawa, Setagaya-ku, Tokyo 156-8506, Japan; ^2^Department of Neurology and Neurosurgery, Montreal Neurological Institute, McGill University, Montreal, QC, Canada H3A 2B4

## Abstract

14-3-3 proteins are abundantly expressed adaptor proteins that interact with a vast number of binding partners to regulate their cellular localization and function. They regulate substrate function in a number of ways including protection from dephosphorylation, regulation of enzyme activity, formation of ternary complexes and sequestration. The diversity of 14-3-3 interacting partners thus enables 14-3-3 proteins to impact a wide variety of cellular and physiological processes. 14-3-3 proteins are broadly expressed in the brain, and clinical and experimental studies have implicated 14-3-3 proteins in neurodegenerative disease. A recurring theme is that 14-3-3 proteins play important roles in pathogenesis through regulating the subcellular localization of target proteins. Here, we review the evidence that 14-3-3 proteins regulate aspects of neurodegenerative disease with a focus on their protective roles against neurodegeneration.

## 1. Introduction

14-3-3 proteins were originally discovered as abundant molecules in the brain [1] and follow-up studies confirmed that the highest tissue concentration of 14-3-3 proteins is in the brain [2]. In fact, 14-3-3 proteins comprise about 1% of total protein from the brain. The role of 14-3-3 proteins has been widely studied because of their remarkable capacity to affect the activity and localization of substrate proteins. In neurons, 14-3-3 proteins function in diverse processes including differentiation, migration, survival, neurite outgrowth, and ion channel regulation [3]. While their neurophysiological functions are not fully understood, 14-3-3 proteins have been implicated in a number of neurological disorders. In this review, we will discuss the evidence that 14-3-3 proteins have a neuroprotective role in the context of neurodegenerative disease.

## 2. General Properties of 14-3-3 Proteins

The 14-3-3 family of adaptor proteins consists of seven isoforms in mammals (*β*, *γ*, *ε*, *η*, *ζ*, *σ*, and *τ*/*θ*) [4]. The family was originally identified and named during a systematic biochemical classification of brain proteins based on their elution number from biochemical fractionation columns [1]. Each family member forms a homo- or heterodimer and binds to target substrates most commonly through phospho-serine/threonine motifs [5, 6]. Dimeric 14-3-3 proteins can bind to two different regions of the same protein to affect the conformation and activity of the substrate [7–9]. A specific protein conformation can be stabilized through 14-3-3 binding or specific phosphorylation sites can be protected through 14-3-3 binding [9]. In addition, 14-3-3 dimers can bind to two different target proteins bringing them into close proximity, leading to a formation of a stable ternary complex [9]. In this way, 14-3-3s are capable of regulating the efficiency of enzymatic activity [10–12]. Further, 14-3-3 proteins regulate the subcellular localization of their substrates to enhance a particular signal or sequester and inhibit a particular pathway [13, 14]. Thus, major molecular functions of 14-3-3 proteins could be summarized as follows: stabilizing specific conformations or modifications, regulating enzyme activity, and regulating subcellular localization.

The occurrence of heterodimers confers an even higher diversity of 14-3-3 functions. The significance of the different isoforms is still not completely understood; however, functions and properties of some isoforms can be proposed. First, specific 14-3-3 isoforms are found in certain diseases. For instance, *η* and *θ* isoform are absent in amyloid plaques in Alzheimer disease, while other isoforms are detected [15]. Second, some 14-3-3 interacting partners bind to 14-3-3 isoforms with significantly different affinities (e.g., c-Raf preferentially binds to 14-3-3 *η* isoforms [10]).

In many cases, the dimerization of 14-3-3 proteins is crucial to their roles as adaptor proteins. However, the role of monomeric 14-3-3 proteins is also an emerging area of interest, particularly when considering the development of therapeutic agents, which may target monomeric 14-3-3 proteins [16]. The presence of 14-3-3 monomers and dimers throughout the cytoplasm, at the plasma membrane, and within intracellular organelles makes this protein family a powerful molecular tool for spatially regulating cell signaling [17–21].

14-3-3 proteins bind to substrate proteins through phosphodependent and phosphoindependent interactions [22, 23]. To date more than 200 proteins have been found to interact with 14-3-3 family members, including protein kinases, receptors, enzymes, structural and cytoskeletal proteins, small G-proteins and their regulators, scaffolding molecules, proteins involved in cell cycle control, proteins involved in transcriptional control of gene expression, and proteins involved in control of apoptosis [9, 24, 25]. This variety of interacting partner underlies the ability of 14-3-3 proteins to participate in such a wide array of cellular and physiological processes.

## 3. 14-3-3 Proteins in Neurological Disorders

14-3-3 proteins play diverse physiological roles and interact with a multitude of substrate proteins during normal development and adulthood [25, 26]. Furthermore, many lines of evidence have identified 14-3-3 proteins as important targets in neuropathological processes [27, 28]. 14-3-3 proteins are detected in the cerebrospinal fluid in various neurodegenerative diseases, such as multiple sclerosis [2, 29], Creutzfeldt-Jakob disease [30–32], and HIV-related neurodegeneration [33]. 14-3-3 proteins also serve as a biomarker of neurological disorders characterized by extensive destruction of neurons in the brain including acute stroke [34] and subarachnoidal hemorrhage [35]. Together, these findings suggest that the presence of 14-3-3 proteins in the cerebrospinal fluid may be indicative of the destruction of brain tissue and leakage of normal cellular proteins into the cerebrospinal fluid. For this reason, 14-3-3 proteins are studied as potential biomarkers of neurodegeneration [36, 37]. In addition, 14-3-3 proteins are found in disease-specific lesions and protein aggregates within the brain, and numerous studies have described 14-3-3 interactions with target proteins that regulate pathogenic processes [3, 27, 28]. This supports the notion that 14-3-3 proteins are involved in the pathogenesis of neurodegenerative disease in addition to their utility as general markers of tissue destruction. Because 14-3-3 proteins could define the subcellular localization of protein substrates, 14-3-3 detection in protein aggregates have encouraged a number of ideas including (1) 14-3-3 proteins may play a protective role through sequestration of toxic pathogenic proteins, (2) 14-3-3 proteins may be sequestered into aggregates resulting in their own loss of function, and (3) 14-3-3 proteins may facilitate formation of protein aggregates that cause a subsequent neurotoxicity. In addition to protein sorting, it is also known that 14-3-3 proteins stabilize their binding partners, protect phosphorylated species of target proteins, and regulate substrate enzyme activity. Failure of these functions could also contribute to neurodegenerative disease. Below, we discuss several specific examples implicating 14-3-3 proteins in the pathogenesis of neurodegenerative diseases.

### 3.1. Parkinson's Disease

Parkinson's disease (PD) is an age-related neurodegenerative disease characterized by loss of dopaminergic neurons in the substantia nigra pars compacta [38]. PD is clinically characterized by progressive rigidity, bradykinesia, tremor, and postural instability [39]. Abnormal protein aggregates called Lewy bodies develop in neurons and are a pathological hallmark of PD. Lewy bodies are present in the brainstem, particularly in the substantia nigra [40, 41]. The main component of Lewy bodies is *α*-synuclein, a regulator of the mitogen-activated protein kinase (MAPK) pathway, which plays an important role in dopamine synthesis [42–44]. Families with gene multiplication of *α*-synuclein exhibit autosomal dominant PD [45] and *α*-synuclein overexpression in an animal model leads to neuronal cell death [46]. Interestingly, expression levels of 14-3-3 *γ*, *ε*, and *θ* were reduced by *α*-synuclein overexpression in an animal model [47]. Conversely, overexpression of 14-3-3 *ε*, *γ*, and *θ* suppressed the aggregation of *α*-synuclein and decreased toxicity induced by rotenone or 1-methyl-4-phenyl-1,2,3,6-tetrahydropyridine (MPTP) [47], two neurotoxins that cause cell death in dopaminergic cells and induce parkinsonian syndrome [48]. These observations are consistent with a model whereby *α*-synuclein could exert its effects at a transcriptional level by reducing the expression of 14-3-3 proteins contributing to pathogenesis in PD.

Immunohistochemical investigation has described intense 14-3-3 staining in Lewy bodies [49]. In particular, *ε*, *γ*, *ζ*, and *θ* isoforms were present, while *β*, *η*, and *σ* were not detectable in Lewy bodies [50]. *α*-synuclein was shown to bind to 14-3-3 proteins and to share amino acid sequence homology with 14-3-3 proteins [51], suggesting that 14-3-3 proteins may be related to *α*-synuclein aggregation. Indeed, 14-3-3 and *α*-synuclein can be coimmunoprecipitated from mammalian brains [52], and coimmunoprecipitation is increased in PD brains [53]. This raises the possibility that interaction between 14-3-3s and *α*-synuclein could have pathogenic roles that are independent of regulating 14-3-3 expression.

**Figure 1:**
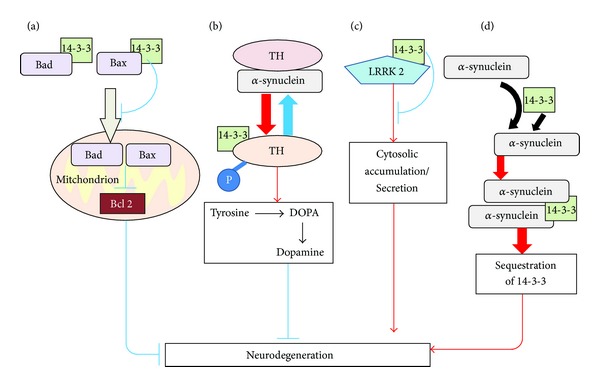
Overview of the molecular basis of the neuroprotective roles of 14-3-3 in Parkinson's disease. 14-3-3 proteins may play a protective role against neurodegeneration in PD by interacting with various proteins. (a) 14-3-3 interacts with Bad and Bax proteins and inhibits their translocation into mitochondria and their subsequent suppression of Bcl-2. These interactions prevent apoptosis of neurons. (b) 14-3-3 proteins interact with phosphorylated tyrosine hydroxylase (TH) to enhance its activity. In contrast, *α*-synuclein binds unphosphorylated TH to reduce its activity. These protein interactions contribute to the maintenance of optimal dopamine levels. Inadequate levels of dopamine lead to a neurodegeneration. (c) 14-3-3 interacts with LRRK2 protein that is related to PD pathogenesis. This interaction may inhibit cytosolic accumulation and secretion of LRRK2. (d) *α*-synuclein interacts with 14-3-3 proteins and forms aggregates, resulting in the sequestration of 14-3-3. This sequestration may contribute to the pathogenesis of PD.

**Figure 2:**
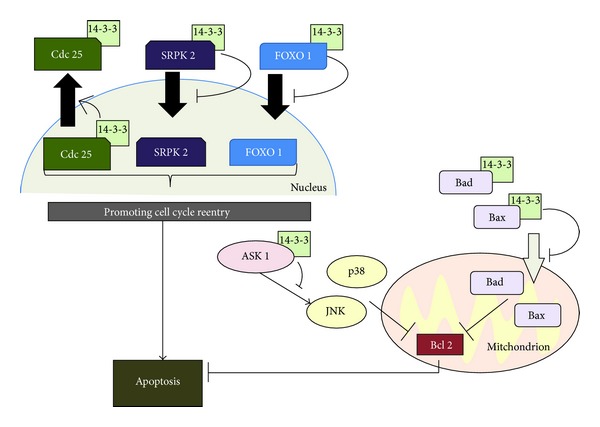
Overview of the molecular basis of 14-3-3 antiapoptotic activity. 14-3-3 proteins inhibit apoptosis through multiple mechanisms including regulating the subcellular localization of pro- and antiapoptotic proteins. 14-3-3 binds to Bad and Bax to sequester them in the cytoplasm. Similarly, SRPK2 and FOXO1 are retained in the cytoplasm through 14-3-3 binding. Cdc25 is exported from the nucleus through a 14-3-3 interaction. SRPK2, FOXO1, and Cdc25 contribute to cell cycle reentry and subsequent apoptosis. Further, the death-promoting activity of ASK1 is antagonized by its binding to 14-3-3 proteins.

There is some evidence that a specific relevant target of 14-3-3 dysregulation may be in neuronal apoptosis in PD (Figures 1(a) and 2). 14-3-3 *θ* interacts with Bad, which is a negative regulator of the antiapoptotic molecule Bcl-2 in mitochondria [54]. 14-3-3 proteins can function as antiapoptotic factors by sequestering Bad protein in the cytoplasm leaving Bcl-2 function unobstructed [54]. Association of 14-3-3 proteins with *α*-synuclein reduced the antiapoptotic activity of Bcl-2 by increasing the levels of Bad protein in mitochondria. 14-3-3 proteins also work as antiapoptotic factors by binding Bcl-2-associated X protein (Bax), which promotes apoptosis upon translocation into mitochondria [55]. 14-3-3 *θ* prevents Bax translocation to the mitochondria and subsequent destruction of the mitochondrial outer membrane. Overexpression of 14-3-3 *θ* reduces dopaminergic cell death in cultured neurons exposed to rotenone and 1-methyl-4-phenylpyridinium (MPP+) and this effect was dependent on binding to Bax, supporting the idea that 14-3-3 proteins are antiapoptotic and may be targeted to promote neuronal survival in PD [56]. Indeed, there is a selective increase in 14-3-3/*α*-synuclein complex formation in the substantia nigra of patients with PD rendering the cell more susceptible to apoptosis [52]. One model is that sequestration of 14-3-3 proteins through interaction with *α*-synuclein results in the loss of appropriate 14-3-3 function contributing to the pathogenesis of PD (Figure 1(d)). The sequestration hypothesis could be relevant to antiapoptotic 14-3-3 substrates and/or other interacting partners with roles in PD pathogenesis.

Additionally, 14-3-3 proteins play a role in the dopaminergic signaling pathway in PD (Figure 1(b)). Dopaminergic neuronal cell death with a reduction of brain dopamine is one of the main features of PD. 14-3-3 *ζ* binds to phosphorylated tyrosine hydroxylase, the rate-limiting enzyme in dopamine synthesis, and leads to prolonged activation of the enzyme [57–59]. *α*-synuclein reduces the activity of tyrosine hydroxylase and subsequent dopamine production through binding to dephosphorylated tyrosine hydroxylase [44]. An intriguing idea is that 14-3-3 proteins may be targeted therapeutically to upregulate dopamine production and ease the progression of dopamine loss in PD.

Another isoform, 14-3-3 *η*, has been shown to bind and negatively regulate parkin, an E3 ubiquitin ligase that is mutated in a familial form of PD [60]. Parkin mediates the targeting of proteins for degradation and its loss of function is thought to result in the accumulation of proteins that are toxic to dopaminergic neurons [53, 61]. The interaction between 14-3-3 and parkin is disrupted when parkin has a mutation that causes autosomal recessive juvenile parkinsonism [53]. Further, *α*-synuclein abrogates 14-3-3 *η*-related parkin inactivation [53]. While it is not clear how these interactions ultimately regulate neuronal parkin activity, these findings suggest potential functional significance for 14-3-3 *η* in PD pathogenesis.

Variants in Leucine-rich repeat kinase 2 (LRRK2) are associated with an increased risk in PD [62]. Although little is understood about its physiological function in PD pathogenesis, it was recently shown to bind to several 14-3-3 isoforms [63]. A common mutation of familial PD in LRRK2 abolished the interaction between 14-3-3 and LRRK2 [64]. Disruption of the interaction between 14-3-3 and LRRK2 led to LRRK2 accumulation within cytoplasmic pools rather than a normal diffuse localization throughout the cell [65]. 14-3-3 proteins might stabilize LRRK2 in the cytoplasm preventing its aggregation. Further evidence suggests that disrupting the 14-3-3-LRRK2 interaction blocks the release of LRRK2 into extracellular microvesicles [66]. A number of lines of evidence suggest that aberrant LRRK2 protein sorting in neurons may contribute to PD pathogenesis (Figure 1(c)). For example, Rab7L1 regulates the intraneuronal sorting of LRRK2 and also *Rab7L1* gene is located in a locus harboring PD susceptibility [67, 68]. Reduced neurite extension in cultured neurons harboring mutant LRRK2 can be rescued by overexpression of Rab7L1 supporting the idea that proper sorting is critical to its function [67]. 14-3-3 proteins may act in a similar fashion to regulate intracellular LRRK2 protein localization.

Together, these studies demonstrate that multiple 14-3-3 isoforms are involved in pathogenic processes of PD, including apoptosis, aberrant dopamine production, and protein sorting.

### 3.2. Amyotrophic Lateral Sclerosis

Amyotrophic lateral sclerosis (ALS) is a rapidly progressing fatal motor neuron disease for which a precise cause has not yet been identified. It is characterized by muscle weakness and atrophy that results in difficulties in breathing, swallowing, and speaking [69]. A neuropathological hallmark of ALS is the presence of intraneuronal neurofilament aggregates [70]. Neurofilament proteins are highly conserved neuronal intermediate filaments characterized on the basis of molecular weight and are composed of a light, medium, and heavy molecular weight isoforms (NF-L, NF-M, and NF-H). The assembly of the filamentous neurofilament complex is dependent on the primary homopolymerization of NF-L, and the stoichiometry of neurofilament subunits is highly regulated [71]. Alterations in the stoichiometry are associated with neurofilament aggregate formation in a variety of transgenic models of motor neuron degeneration [72]. NF-L mRNA levels are selectively reduced in degenerating spinal motor neurons in ALS patients [73] and this may be of specific relevance to the genesis of neurofilament aggregates in ALS. NF-L mRNA are stabilized through binding with several proteins, such as 14-3-3, TAR DNA binding protein (TDP-43) and copper/zinc superoxide dismutase (SOD) [74, 75]. In both familial ALS and an ALS animal model, 14-3-3 *β* and *γ* are present in Lewy body-like hyaline inclusions (LBHI) [76]. TDP-43, and SOD were also identified as a component of LBHI [77, 78]. Together, this supports a model whereby the formation of LBHI could sequester proteins that favor the stabilization of NF-L mRNA, leading to neurofilament aggregates in ALS. Furthermore, recent work revealed that 14-3-3 interacts with NF-L in an NF-L-phosphorylation-dependent manner and diminished 14-3-3 interaction with NF-L resulted in the formation of neurofilament aggregation [79]. The data is consistent with a central role of 14-3-3 proteins in quality control of neurofilaments through regulating stoichiometry and preventing aggregate formation in ALS.

Other studies have shown that mRNA for 14-3-3 *ζ* and *θ* are upregulated following hypoglossal nerve injury in rats and that 14-3-3 *θ* is upregulated in the spinal cord of ALS patients [80, 81]. Proteomic analysis from spinal cord tissue from ALS patients identified elevated expression and/or activation of many protein kinases, including protein kinase C (PKC), glycogen synthesis kinase 3*β* (GSK3*β*), calcium-calmodulin-dependent protein kinase kinase (CAMKK), Akt, S6 K, and protein kinase A (PKA), which may augment neural death in ALS [82]. An intriguing idea is that upregulated 14-3-3 proteins could function to sequester hyperphosphorylated substrates or kinases, attenuating the effects of elevated kinases and playing a neuroprotective role in ALS. For example, 14-3-3 *β* and *γ* are able to interact with PKA subunits and inhibit PKA activation [83].

### 3.3. Alzheimer Disease

Alzheimer disease (AD) is a common form of progressive dementia neuropathologically characterized by cortical and perivascular amyloid plaques and neurofibrillary tangles (NFTs). NFTs are composed of paired helical filaments with the microtubule-associated protein tau as a main component [84]. Tau, a major microtubule-associated protein in neurons, binds and stabilizes microtubules. Tau phosphorylation reduces its affinity for microtubules and tau is hyperphosphorylated in AD. Phosphorylation is thought to cause loss of tau function, instability of the microtubule, formation of NFTs, and neurodegeneration [84, 85]. 14-3-3 proteins have been detected in NFT of AD patients with 14-3-3 *ζ* being the most highly immunoreactive [15]. Further study has demonstrated that 14-3-3 *ζ* facilitates GSK3*β*-dependent phosphorylation of tau by enhancing the affinity of GSK3*β* for tau [86, 87]. It is also noteworthy that proteins bound to 14-3-3 *ζ* are relatively resistant to protein phosphatases, raising the possibility that 14-3-3 *ζ* may enhance the strength or duration of kinase-dependent signals in pathogenic circumstances [87]. However, it has also been shown that 14-3-3 *β*, *η*, and *ζ* bind with high affinity to tau that has been phosphorylated by Akt and PKA [88] and this interaction reduced the aggregation of tau *in vitro*. On the contrary, tau phosphorylated by GSK3*β* rapidly aggregated [88]. Thus it appears that 14-3-3 proteins can have dual roles in tau aggregation and their global effects are likely to be context dependent.

Reactivation of the cell cycle in AD neurons is a potential mechanism that drives cells towards neuronal atrophy [89–91]. There is some evidence that 14-3-3 proteins may play a neuroprotective role through this molecular pathway. There is evidence that activity of the cell cycle kinase Cdk5 and the cell cycle phosphatase Cdc25 are increased in clinical AD samples [92, 93]. Inhibition of Cdk5 or Cdc25 promoted neuroprotection in cultured neurons treated with an oligomeric amyloid *β* 1–42 (A*β*42), one of toxic species in AD brain [94]. Intriguingly, 14-3-3 *ε* binds Cdc25 and sequesters it in the cytoplasm, and A*β*42 diminished this interaction [94]. When the interaction between Cdc25 and 14-3-3 *ε* is inhibited through Cdk5-mediated phosphorylation of Cdc25, neurotoxicity and neuronal death are promoted in AD [94] (Figure 2).

Serine-arginine protein kinase 2 (SRPK2) is a cell cycle-regulated protein kinase, which can translocate into the nucleus and promote cyclin D1 upregulation and cell cycle reentry [95] (Figure 2). 14-3-3 *β*, *ε*, and *σ* interact SRPK2 and inhibit its translocation into the nucleus [95]. In an AD mouse model, depletion of SRPK2 in the dentate gyrus alleviated impaired cognitive behaviors and defective LTP [96]. Together, these observations support the idea that 14-3-3 proteins can suppress the functions of many critical substrates that have been implicated in cell cycle reentry of neurons in AD.

Finally, 14-3-3 proteins may directly regulate apoptotic pathways in AD. Active Cdk1 promotes neuronal death in postmitotic granule neurons by phosphorylating the forkhead box, group O 1 (FOXO1) transcription factor upon depolarization [97]. FOXO1 is also a binding partner of 14-3-3 *β* [97]. Phosphorylated FOXO1 dissociates from 14-3-3 and translocates into the nucleus where FOXO1-dependent transcription induces neuronal cell death [97]. This finding also suggests that 14-3-3 proteins play a positive role in promoting neuronal cell survival and could protect against AD pathogenesis; however, direct experiments to test this hypothesis in an AD model remain to be done (Figure 2).

### 3.4. Creutzfeldt-Jakob Disease

Creutzfeldt-Jakob disease (CJD) is a neurodegenerative disorder clinically characterized by a rapidly progressive dementia and a characteristic combination of neurological features [98, 99]. Classic CJD occurs sporadically through the transformation of prion proteins into abnormal species. The definite diagnosis of CJD can only be made histopathologically; thus, the identification of diagnostic markers for CJD is an unmet need. Several studies confirmed that 14-3-3 proteins from cerebrospinal fluid (CSF) may be reliable markers for CJD [30, 31]. The total content of 14-3-3 protein was significantly higher in the CSF of patients with definite or probable CJD than in patients with other neurodegenerative disorders [100]. Extensive destruction of neurons in brain in CJD may result in leakage of 14-3-3 proteins into the CSF, but the specific link to CJD also raises the possibility that 14-3-3 proteins play specific roles in the pathogenesis of CJD. 14-3-3 *ζ* was detected in amyloid plaques of sporadic and variant CJD, while other isoforms were absent from these deposits [101].

### 3.5. Polyglutamine Disease

Spinocerebellar ataxia type 1 (SCA1) is a lethal neurodegenerative disorder caused by expansion of a polyglutamine (poly-Q) tract in ataxin-1 [102]. A prominent site of pathology in SCA1 is cerebellar Purkinje neurons where mutant ataxin-1 enter the nucleus and aggregates in inclusion bodies (IB) [103, 104]. While wild type ataxin-1 shuttles back and forth between nucleus and cytoplasm, ataxin-1 with a poly-Q tract fails to properly transport back to the cytoplasm after entry into the nucleus [105]. Although nuclear IB were identified as a pathological hall mark primarily in the affected brain regions, several studies suggest that the function of IB is neuroprotective [106, 107]. 14-3-3 *β*, *ζ*, and *ε* proteins bind to phosphorylated ataxin-1 and impede its translocation into the nucleus, prevent its dephosphorylation, and stabilize the protein [108]. 14-3-3 could therefore be playing dual functions on SCA1 pathogenesis. 14-3-3 interactions with ataxin-1 may play a positive role in inhibiting ataxin-1 translocation to the nucleus or it could play a negative role by stabilizing ataxin-1 harboring a poly-Q tract. The finding that a partial loss of 14-3-3 *ε* improves cerebellar phenotypes in SCA1 suggests that 14-3-3 contributes to the pathogenesis of SCA1 [109].

Huntington's disease (HD) is an autosomal dominant progressive neurodegenerative disorder caused by poly-Q expansions in the Huntingtin protein [110]. 14-3-3 *ζ* might scavenge misfolded Huntingtin proteins by facilitating the formation of aggregates possibly for neuroprotection [111]; these aggregates are referred to as IBs in Huntington's disease. Huntingtin with a poly-Q tract also forms IB, but 14-3-3 reduction by siRNA abolished Huntingtin IB formation [112], suggesting 14-3-3 participates in Huntingtin IB formation.

### 3.6. Excitotoxicity Dependent Neurodegeneration

Temporal lobe epilepsy is a common type of human epilepsy characterized by extensive loss of hippocampal pyramidal neurons and associated gliosis. Frequent seizures lead to neuronal cell death, which may be caused by the apoptotic cell death pathway [113]. The subcellular distribution and expression levels of 14-3-3 proteins are also regulated by seizure activity raising the possibility that antiapoptotic 14-3-3 functions could be disrupted by seizures [114, 115].

Apoptosis signal-regulating kinase 1 (ASK1) is mitogen-activated protein kinase kinase kinase (MAPKKK), which plays a pivotal role in cell apoptosis [116]. ASK1 is weakly expressed in the hippocampus from control brains but is enhanced 4–24 hr after seizures [117]. 14-3-3 *β* binds and inhibits the activity of ASK1 [117]. Notably, ASK1 dissociates from 14-3-3 protein after seizures [117]. Although the precise mechanism by which 14-3-3s may regulate ASK1 remains to be determined, it is reasonable to speculate that dissociation of the 14-3-3-ASK1 complex promotes apoptosis.Further, reduction of 14-3-3 expression leads to activation of Bad protein after the seizure, further demonstrating how a pathological condition may circumvent the antiapoptotic role of 14-3-3 proteins [117] (Figure 2).

Ischemic injury in the central nervous system causes neurodegeneration by neuronal apoptosis. A recurring theme in this paper is the role of 14-3-3 interactions with proapoptotic proteins in preventing neuronal apoptosis. After ischemia, Bad and Bax dissociated from 14-3-3 *ζ* and translocated to the mitochondria to suppress the antiapoptotic protein Bcl-2 [118, 119]. Interestingly, a brief episode of sublethal ischemia followed by reperfusion prevents the lethal effect of subsequent periods of prolonged ischemia [120] and ischemic preconditioning limits 14-3-3-ASK1 dissociation and subsequent apoptotic signaling [121].

The same antiapoptotic function of 14-3-3 proteins has been reported in retinal ganglion cells (RGC) in a glaucoma model in rat. Elevated intraocular pressure leads to enhanced expression of 14-3-3 *β*, *ζ*, *η*, *γ*, and *θ* in RGCs [122]. Affinity pull-down analysis demonstrated that 14-3-3 proteins are associate with Bad in RGCs [122]. Thus, 14-3-3s may sequester Bad to the cytoplasm of RGCs, playing a neuroprotective role in experimental glaucoma.

### 3.7. Glia-Mediated Neurodegeneration

Multiple system atrophy (MSA) is a fatal multisystem progressive disorder characterized clinically by various combinations of autonomic failure, cerebellar symptoms, parkinsonism, and pyramidal signs [123]. Glial cytoplasmic inclusion (GCI) is a distinctive hallmark for MSA [124, 125]. The GCI is immunopositive for *α*-synuclein [126, 127] and is predominantly found in oligodendrocytes [124, 125]. Accumulation of *α*-synuclein might be causally linked to the progression of MSA, because *α*-synuclein aggregation in oligodendrocytes may cause demyelination, resulting in initiation of neurodegeneration [128]. 14-3-3 proteins may play a role in *α*-synuclein accumulation in GCI. Immunohistochemical analyses revealed that both *α*-synuclein and 14-3-3 proteins are colocalized in the GCIs in MSA brains [129]. Similar to the PD pathogenesis, the interaction between 14-3-3 and *α*-synuclein may have a role in an aggregation of *α*-synuclein in oligodendrocytes, resulting in development of MSA.

Multiple sclerosis (MS) is a chronic inflammatory disease of the CNS of autoimmune origin [130]. Although many aspects of MS pathogenesis have been elucidated, exact causal mechanisms are still not fully understood. Levels of 14-3-3 proteins have been shown to be increased in glial cells of MS patients [131]. In particular, 14-3-3 *β*, *ε*, *ζ*, and *η* are intensely expressed in reactive astrocytes in the demyelinating lesions of MS [131]. Protein overlay analyses and coimmunoprecipitation experiments showed that 14-3-3 *β*, *ε*, and *ζ* isoforms interact with vimentin and glial fibrillary acidic protein (GFAP) in astrocytes. Because these glial intermediate filament proteins are copolymerized in assembled filaments in reactive astrocytes [132, 133], 14-3-3 protein may act as a bridge between vimentin and GFAP or as a bundle of vimentin with GFAP in the same filament. As reactive astrocytes release glutamate or proinflammatory cytokines to cause neuronal death [134, 135], 14-3-3 proteins may contribute to the MS pathogenesis through stabilization of cytoskeletal networks in reactive astrocytes.

## 4. Conclusion and Perspective

14-3-3 involvement in multiple cellular processes has prompted investigators to study their role in various pathological processes. Investigation into the numerous proteins and pathways that 14-3-3 proteins regulate is ongoing. Changes in 14-3-3 protein expression levels in the CNS and CSF of patients with neurodegenerative disease has spurred investigation into their utility as biomarkers and into their potential roles in specific pathologies.

Numerous studies suggest that 14-3-3 is involved in disease-associated apoptosis and pathologies in a variety of conditions including PD, ALS, AD, epilepsy, CJD, MSA, and MS. Isoform-specific histology and involvement in each disease mentioned in this review are summarized in Table 1. Localization of specific 14-3-3 isoforms in protein aggregates suggest that 14-3-3 proteins participate in aggregate formation or that their normal protective functions may be disrupted through such aggregation. Antiapoptotic functions of 14-3-3 proteins through regulation of Bad and Bax protein translocation could play important neuroprotective roles. Thus, defining of subcellular localization of 14-3-3 interacting partners may be a major role of 14-3-3 proteins in the pathophysiological process of neurodegeneration.

The potential involvement of 14-3-3 proteins in various neurodegenerative diseases encourages future investigation to elucidate how 14-3-3s impact pathologies that are unique and common to each disease. Future studies will reveal relevant binding partners of 14-3-3 proteins in specific pathologies and may aid in the conception and validation of therapeutic targets to treat neurodegenerative conditions.

## Figures and Tables

**Table 1:** 14-3-3 isoforms in neurodegeneration.

14-3-3 isoform	Isoform-specific histology	Interacting partner and disease involvement
*β*	Tangles in AD LBHI in ALS GCI in MSA	LRRK2; PD NFL mRNA; ALS Tau, SPRK2, FOXO1; AD Ataxin-1; SCA1 ASK1; Epilepsy GFAP; MS

*γ*	Tangles in AD Lewy bodies in PD LBH in ALS GCI in MSA	LRRK2; PD NFL mRNA; ALS

*ε*	Tangles in AD Lewy bodies in PD GCI in MSA	LRRK2; PD Cdc25, SPRK2; AD Ataxin-1; SCA1 GFAP; MS

*ζ*	Tangles in AD Lewy bodies in PD Amyloid plaque in CJD GCI in MSA	Tyrosine hydroxylase, LRRK2; PD NFL mRNA; ALS GSK3*β*, tau; AD Ataxin-1; SCA1 Huntingtin; HD Bad, Bax; Ischemia GFAP; MS

*η*		Parkin, LRRK2; PD NFL mRNA; ALS Tau; AD

*σ*	Tangles in AD	SPRK2; AD

*θ*/*τ*	Lewy bodies in PD GCI in MSA	Bad, Bax, LRRK2; PD NFL mRNA; ALS
